# A single‐cell census of pulmonary endarterectomies tissue across different anatomical regions in chronic thromboembolic pulmonary hypertension

**DOI:** 10.1002/ctm2.70864

**Published:** 2026-10-11

**Authors:** Rui Zhang, Yunlong Zhang, Huiting Li, Jing Zhang, Shou Han, Xinbo Cheng, Qinhua Zhao, Jing He, Cijun Luo, Hongling Qiu, Jianfeng Zhang, Sugang Gong, Lan Wang

**Affiliations:** ^1^ Department of Cardio‐Pulmonary Circulation Shanghai Pulmonary Hospital, Tongji University School of Medicine Shanghai China; ^2^ Department of Biological Sciences and Technology, College of Biological Science and Medical Engineering Donghua University Shanghai China; ^3^ Department of Cardiovascular Surgery, School of Medicine Shanghai Chest Hospital, Shanghai Jiao Tong University Shanghai China

**Keywords:** chronic thromboembolic pulmonary hypertension, distal remodelling, proximal occlusion, pulmonary endarterectomies tissue, single‐cell RNA sequencing

## Abstract

**Background:**

Pulmonary endarterectomy (PEA) remains one of potentially curative options for chronic thromboembolic pulmonary hypertension (CTEPH). The operation can remove proximal thromboembolic materials and their secondary branches. However, the cellular landscape and pathological features across different anatomical regions remain limited understood.

**Methods:**

Ten patients with CTEPH undergoing PEA surgery and six reference distal pulmonary arteries were conducted single‐cell transcriptomics to profile the cellular composition of the main and its bifurcate portion.

**Results:**

Pronounced muscularised and fibrotic thrombi organisation were presented in the main portion, and the proportion of mononuclear phagocytes was significantly increased. Inflammatory response and pro‐inflammatory activity were attributed to CD8+ effector T cells and nature killer cells subsets and a M1‐like (high IL1B and TNF, minimal IL6) phenotypic macrophage subcluster. The colander‐like lesions were dominant in the bifurcate portion, and the percentage of plasma cells was markedly increased. Immune dysregulation was induced from CD4 naïve T cells, and a downregulation of M2‐like macrophage infiltration into the vascular wall. The expression of plasminogen activator, urokinase receptor, urokinase receptor (PLAUR; plasminogen activator) were observed in M2‐like (high MRC1 and CD163, virtually no IL1B or TNF) macrophage in bifurcate portion. Notably, one of smooth muscle cells (SMCs) subclusters in the bifurcate portion gained more significantly myofibroblast markers than those in the main portion, and other subclusters remained the function of SMCs.

**Conclusions:**

The regions with colander‐like lesions changes had more obviously T cell and macrophages driving immune‐inflammatory response and SMCs differentiation, suggesting that combining therapeutic interventions after surgical removal of accessible thrombus may be beneficial.

**Key points:**

- This study reveals the comprehensive cellular composition in main/lobar and bifurcate pulmonary endarterectomy (PEA) specimens at the single‐cell level. The bifurcate regions with colander had more obviously T cell and macrophages driving immune‐inflammatory response and smooth muscle cells (SMCs) differentiation, however, the remodelling of distal or micro‐vessels still remained.

## Graphical abstract

This study provides the first intra‐individual, regional single‐cell comparison of main/lobar versus bifurcate pulmonary endarterectomy (PEA) specimens.

It reveals distinct macrophage polarisation, divergent T‐cell compositions and pronounced smooth muscle cell (SMC)‐to‐myofibroblast transition in the bifurcate region.

These findings highlight regional cellular heterogeneities, offering cellular evidence for why complete distal endarterectomy is critically important.

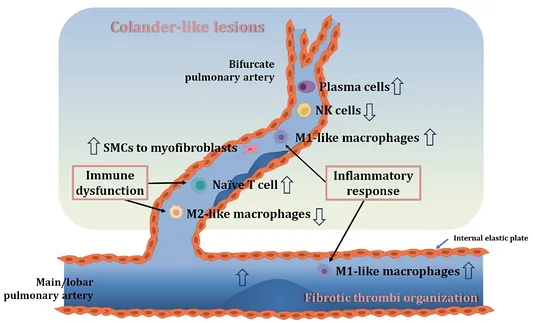

## 1 INTRODUCTION

Chronic thromboembolic pulmonary hypertension (CTEPH) was recognised as a subtype of pulmonary hypertension (PH) due to pulmonary arterial obstruction.^1^, ^2^, ^3^ The unresolved thromboembolism and its pathophysiology warrant further investigation. CTEPH is a chronic progressive condition caused by small vascular remodelling.^3^, ^4^ The surgical removal of accessible pulmonary artery (PA) lesions by pulmonary endarterectomy (PEA) has been the state‐of‐the‐art treatment option.^1^, ^5^ This procedure is started more proximally in the plane of intima, then carefully followed until the thickened fibrous tissue with distal segmental and subsegmental branches is reached.^1^, ^6^ According to a surgical classification proposed by the group at the University of California, San Diego, Level I and II disease is more favourable for surgical candidacy.^6^ Therefore, a complete endarterectomy could clear the hidden obstructive material located at the far distal tail ends of vascular tree. However, the cellular landscape and pathological features behind thrombofibrotic occlusions remain limited understanding.

In the case of CTEPH, the chronic clots are yellow, adhering to the pulmonary vascular wall and contain collagen, elastin and inflammation cells.^7^, ^8^ The distal ‘closed’ arterial tree are spared from high pressure, whereas the ‘open’ arterial tree is exposed to high pressure, displaying pathological characteristics similar to pulmonary arterial hypertension (PAH).^9^, ^10^, ^11^ Intriguingly, a study from Japanese reported that the different histopathology in PEA specimens, with central recognisable lesions exhibited fresh thrombus and early phase of organisation, while the bifurcate lesions showed colander‐like lesion.^12^ In this study, undifferentiated smooth muscle cells (SMCs) and vasoconstrictor endothelin were more abundant in the central than bifurcate lesions. Although the heterogeneous cell populations have been identified in several studies, there is a lack of information on cellular differences between proximal and their bifurcate lesions.^13^, ^14^, ^15^ For example, Miao et al. identified comparable SMCs, myofibroblasts and immune cells subpopulations in CTEPH thrombus via pseudotime analysis; However, the notable proportion (∼20%) of unidentified cells, potentially due to doubles, mitochondrial contamination or database driven annotation.^14^, ^15^ Additionally, although Viswanathan et al. successfully delineated relevant subclusters, the information was limited in bifurcate portion.^13^

Using single‐cell RNA sequencing (scRNAseq), we can identify the key pathological cells type in CTEPH. Here, the samples of CTEPH fibrothombotic and their bifurcate material removed at the time of PEA surgery were used. We also used healthy PA samples as reference PAs to provide general context for cell‐type annotation and marker expression. The findings of this study provide novel insights into the altered cellular landscape and progression of pathological remodelling based on the different anatomical regions.

## 2 RESULTS

### 2.1 Single‐cell atlas of CTEPH tissues across anatomical regions

We profiled 60 019 single cells from main portion, 60 627 from its bifurcate portion and 36 733 from reference PAs (Figure 1A,B). Based on annotations from the Human Primary Cell Atlas, we identified 25 raw clusters (Figure S1). These 25 clusters comprise nine distinct cell populations, including endothelial cells (ECs), SMCs, fibroblasts, T and nature killer (NK) cells, B cells, mast cells, plasma cells, proliferating cells and mononuclear phagocytes (MPs, including macrophages, monocytes, conventional type 1 and 2 dendritic cells and neutrophils; Figure 1C). Compared to reference PAs, the fraction of ECs, SMCs, fibroblasts and mast cells were significantly decreased in CTEPH, while plasma cells were increased (Figure 1D). The main portion contained a higher proportion of MPs (66% vs. 54%), but lower proportions of ECs (1% vs. 3%), SMCs (9% vs. 15%), and plasma cells (3% [1819 cells] vs. 6% [3601 cells]) compared to the bifurcate portion (Figure 1E). Cell‐type‐specific gene expression pattern were well‐differentiated (Figures 1F and S2). To assess the gene–disease associations in the single‐cell transcriptome, we examined the cell‐type‐specific expression patterns of genes associated with Mendelian disorders. Based on the Online Mendelian Inheritance in Man (OMIM) dataset, ECs exhibited high expression of the *CAV1* and *ENG* gene in CTEPH, which are known to mutate in PAH (Figure 1G). MPs highly expressed *FCGR2A* and *F13A1* genes, mutations of which can linked to cystic fibrosis and thrombophilia due to thrombin deficiency. Additionally, *PROS1* gene was enriched in fibroblasts, suggesting a role in thrombophilia due to protein deficiency (Figure 1G). In CTEPH, predicted cell–cell interactions between SMCs and fibroblasts were significantly increased compared to reference PAs. Interactions between MPs and T and NK cells were predominant in the bifurcate portion (Figure S3).

**FIGURE 1:**
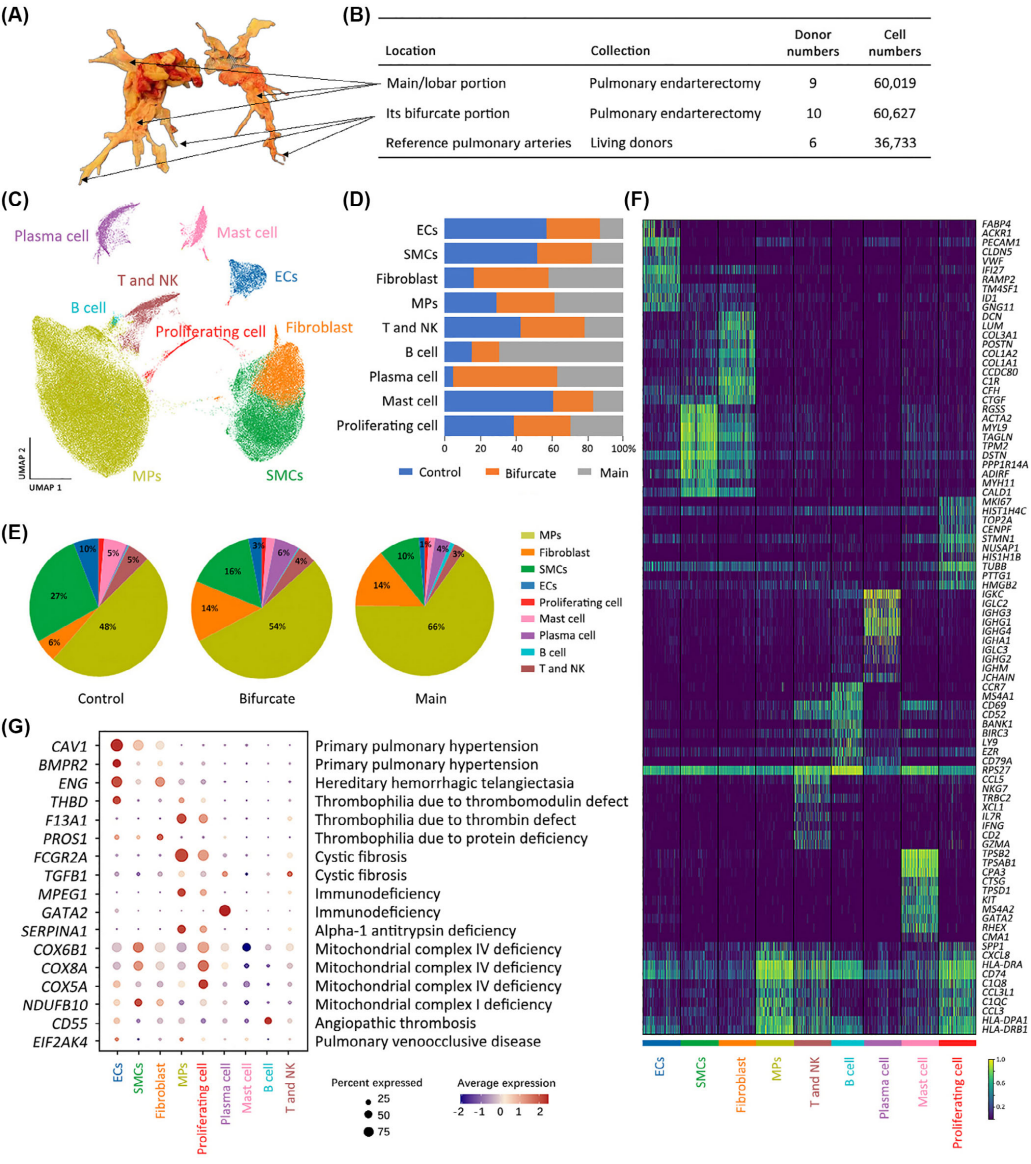
Cell census identifies zonation of pulmonary endarterectomised tissues in patients with chronic thromboembolic pulmonary hypertension and healthy pulmonary arteries. (A) Schematics of anatomical regions analysed in this study. (B) Table providing details of the anatomical regions, donors and cell numbers presented in this figure. (C) Uniform Manifold Approximation and Projection (UMAP) depicting the major clusters present in main portion, its bifurcate portion and references’ pulmonary arteries. (D) Horizontal slice bar representing the anatomical distribution of each identified cell type. (E) Pie charts displaying the cellular composition of each anatomical region. (F) Heat map depicting the average expression levels per cluster of the top differentially expressed markers in each cluster. (G) Dot plot demonstrating gene expression levels and percentage of cells expressing genes associated with specific lung phenotypes based on the Online Mendelian Inheritance in Man (OMIM) database. All panels depict the number of cells and individuals described in (B). MPs, mononuclear phagocytes.

Histological changes were evident in PEA sample between the bifurcate and the main portion (Figures 2 and S4). The main portion exhibited a pronounced presence of muscularised and fibrotic thrombi organisation, while the bifurcate portion showed colander‐like lesions and recanalised vascular lesions (Figure 2A). Masson staining indicated that collagen fibres (blue) predominantly filled thrombi in the main portion, whereas collagen co‐existed with smooth muscle fibres (pink) in the bifurcate region (Figure 2B). Additionally, the main portion contained abundant elastic fibres (black), collagen fibres (pink), hemocytes and myofibres (yellow), while collagen fibres were more prevalent in the bifurcate portion (Figure 2C). Immunofluorescence staining revealed higher expression of fibroblasts in the main portion (Figure 2D). Conversely, in the bifurcate region, fibroblast and SMCs were noted (Figure 2E).

**FIGURE 2:**
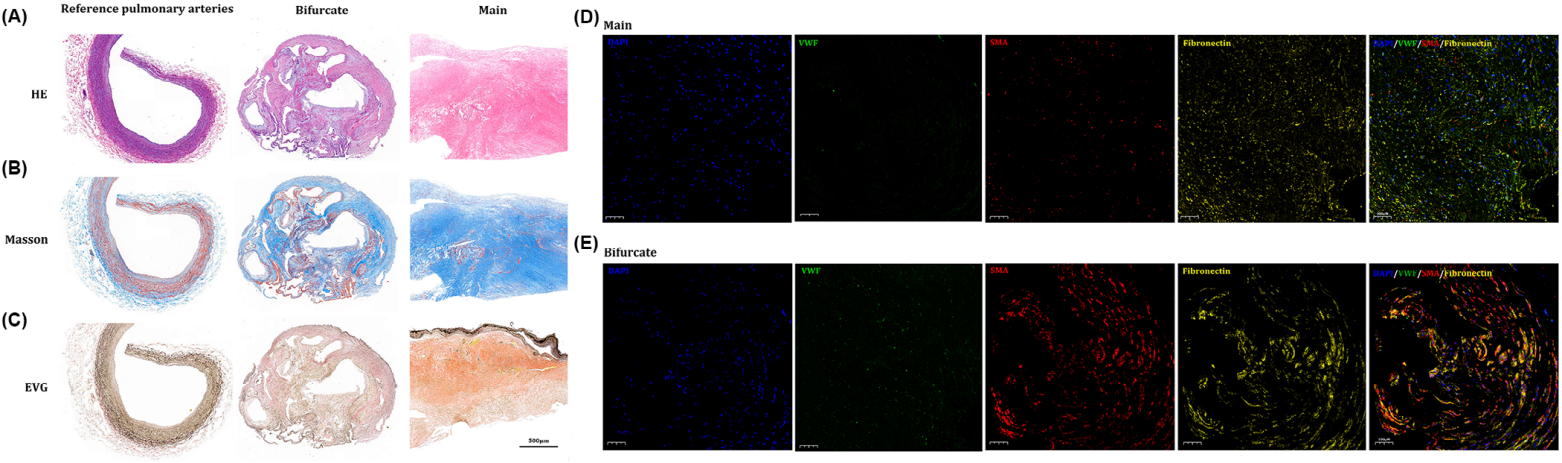
Histopathological finding of pulmonary endarterectomised tissues in the main portion, bifurcate portion and reference pulmonary artery. (A) Representative image of haematoxylin and eosin staining. (B) Representative image of Masson staining, including collagen fibre (blue), smooth muscle cell (pink) and haemocyte (red). (C) Elastica van Gieson staining represents the elastic fibre (black), collage fibre (pink) and haemocyte and myofibre (yellow). Immunofluorescence with markers for endothelial cells (vWF), smooth muscle cells (α‐SMC actin) and fibroblasts (fibronectin) (D) in the bifurcate portion and (E) the main portion.

### 2.2 Molecular features of immune compartments in CTEPH tissues

To further describe immune compartments in CTEPH, we analysed 45 289 single cells from the main portion, and 41 010 from its bifurcate portion (Figure 3A). Six major cell types were identified: T and NK cells, B cells, MPs (macrophages, monocytes, cDC1 and cDc2 subcluster), mast cells, plasma cells and proliferating cells (Figure 3B). These populations were well distinguished by their top marker expression (Figure S5) and cluster‐defining differentially expressed genes (DEGs; Figure S6). The proportion of T and NK cells were markedly decreased but an increase in MPs in the main portion compared to the bifurcate portion (Figure 3C). The cell transcriptional phenotypes altered in both regions, with significant upregulation of inflammatory response genes in MPs (*CXCL8* and *IL1B*), inhibitory markers of NK cell‐mediated cytotoxicity (*KLRB1*) and inflammatory mediators in T and NK cells (*IL7R*), along with CCR7 in B cells, compared to reference PAs (Figure 3D). Conversely, the main portion, there are a significantly increased gene in *APOE* and *CTSB* in MPs, and *CPA3* in mast cells, which are associated with lipid transport and degradation of endogenous proteins, compared to the bifurcate portion (Figure 3D). Trajectory inference analysis illustrated the pseudo‐temporal ordering of these six cell types, with two distinct branching points at different states (Figure S7). In the main portion, proliferating cells was notably enriched at one end, whereas T and NK cells remained stable at the corresponding end in the bifurcated portion (Figure 3E). The percentage of plasma cells increased at another end in the main portion. Furthermore, Genome‐Wide Association Studies (GWAS) analysis linked specific immune‐related genes to CTEPH susceptibility, including *HLA‐DPA1* and *DNAJC10* (Figure 3F).

**FIGURE 3:**
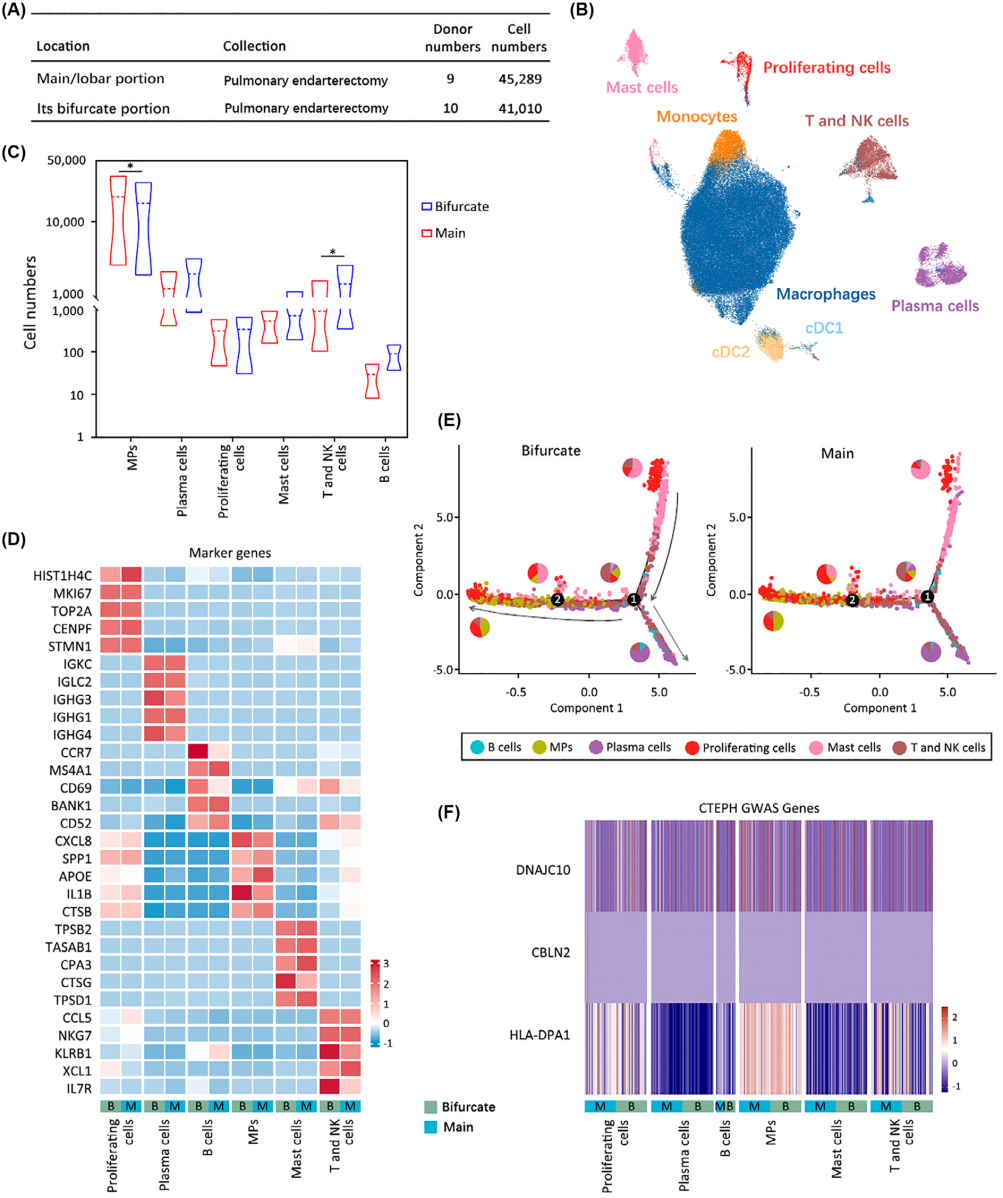
Distinct immune compartments programs in pulmonary endarterectomised tissues of patients with chronic thromboembolic pulmonary hypertension (CTEPH). (A) Overview table with cell numbers for the main and bifurcate portions. (B) Uniform Manifold Approximation and Projection (UMAP) displaying major immune cells analysed coloured by their specific cluster assignment. (C) Violin plots depicting cell numbers of the bifurcate and main portions in the major cluster. (D) Heat map displaying the top five differentially expressed genes per cluster in the main and bifurcate portions. (E) Pseudotime developmental trajectory analysis depicting the relationship between major immune clusters. (F) Heat map displaying the expression of CTEPH‐associated genes from Genome‐Wide Association Studies (GWAS). Only genes present in our list of differentially expressed genes are depicted for each cell cluster. The significance analysis in panel (C) was calculated using Fisher's exact test, corrected for multiple comparison using the Bonferroni method and * represented *p* <  .001. All the differential expression analyses in panels (D) and (F) were performed using the non‐parametric two‐sided Wilcoxon rank‐sum test in Seurat.

### 2.3 Heterogeneous populations of T and NK cells in CTEPH samples

We identified four immune cell clusters: CD4 naïve T cells, CD8 naïve T cells, CD8 effector T cells (Teff), NK cells and NKT cells (Figure 4A). The expression of key genes is shown in Figures S8 and S9. In reference PAs, CD8 Teff and NK cells were the dominant populations, whereas CD8 naïve T and CD4 naïve T cells were significantly increased in CTEPH. In the bifurcate portion, NK cell proportions were reduced, whereas NK cells were maintained in the main portion (Figure 4B). In CTEPH, NK cells and CD8 Teff subsets exhibited elevated expression of cytokines and chemokines, such as XCL2, IL32, ANXA1 and ISG15 (Figure 4C). Notably, CD8 Teff cells in CTEPH lacked metallothionein genes (*MT1E*, *MT2A*) and granzymes (*GZMH*, *GZMB*), while showing increased expression of *CCR7* (Figure 4D). In the main portion, CD8 naïve T cells displayed elevated expression of immunoglobulin genes (*IGKC*, *IGHG1*, *IGHG4*, *IGLC2*). Additionally, NK cells in CTEPH showed reduced expression of *NCR1* and *KLRC1* (Figure 4D). In the main portion, CD4 naïve T cells exhibited increased expression of *TXNIP*, and CD8 naïve T cells showed elevated expression of *IL32*. Conversely, CD8 Teff cells had reduced expression of *MGP*, and NK cells showed lower expression of *NKG7* (Figure 4E). Among cytokine‐related ligand–receptor pairs, TNF–TNFRSF and FAS–FASLG interactions were prominent in both reference PAs and CTEPH. In the main portion, the number of these ligand–receptor pairs was significantly reduced. In the bifurcate portion, NK cells communicated exclusively with CD4 naïve T cells via TNFSF14 and TNFRSF1A interactions (Figures 4F and S10).

**FIGURE 4:**
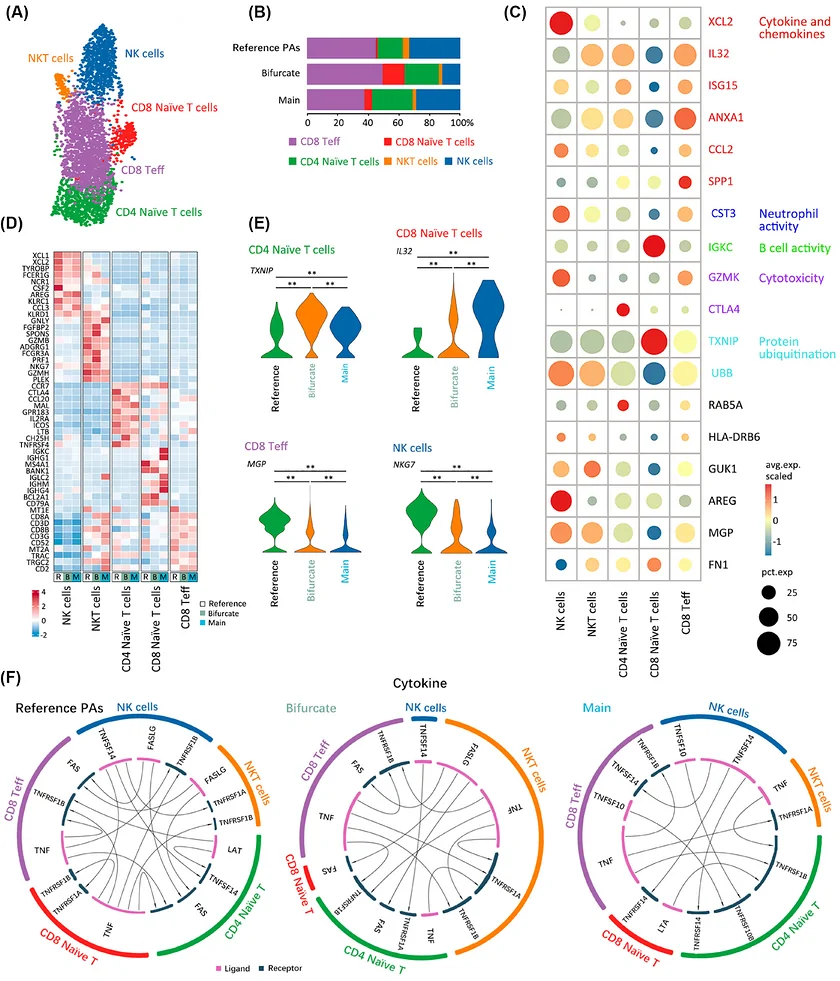
Characterisation of T and NK cells in pulmonary endarterectomised tissues from patients with chronic thromboembolic pulmonary hypertension and healthy pulmonary arteries. (A) Uniform Manifold Approximation and Projection (UMAP) displaying the T and NK cells clusters present in main portion, its bifurcate portion and references’ pulmonary arteries. (B) Horizontal slice bar representing the anatomical distribution of each identified cell type. (C) Dot plot showing the average expression per cluster of genes differentially expressed in T and NK cell populations. Gene names coloured according to functional categories. (D) Heat map displaying gene expression levels of the top 10 differentially expressed genes in each cluster. (E) Violin plots of illustrating selected differential genes in T and NK cells between patients and reference PAs. Significance was calculated using the non‐parametric two‐sided Wilcoxon rank‐sum test in Seurat. An asterisk (*) represents the *p*  <  .001. (F) Circo plots depicting the cytokine type ligand–receptor interactions among different cell types.

### 2.4 Macrophages with an inflammatory profile in the bifurcate portions

Seven distinct macrophage subclusters were identified based on their top marker expression (Figure 5A). Notably, macrophage subcluster 2 exhibited high expression of *IL1B* and *TNF*, with minimal *IL6* expression, indicating an M1‐like macrophage profile. In contrast, macrophage subcluster 1 showed high expression of *MRC1* and *CD163*, with virtually no expression of *IL1B* or *TNF*, suggesting an M2‐like macrophage phenotype (Figure 5B). In the main portion, the proportion of M1‐like macrophages (subcluster 2) was significantly increased, while M2‐like macrophages (subcluster 1) were decreased (Figure 5C). In both bifurcate and main portions, M1‐like macrophages displayed significantly elevated expression of *FN1*, *VCAN* and *TIMP1*, while *OLR1*and *IL1B* were significantly decreased. In contrast, M2‐like macrophages in the bifurcate portion showed higher expression of *PLAUR* and *SELENOP* (Figure 5D and Table S1). Pseudotime analysis and BEAM revealed an upregulation of an inflammatory profile, including CXCL1/2/8, CCL3, IL1B and CCL3L1, towards cell fate 2 (Figure 5E). HALLMARK database identified significant upregulation of pathways related to inflammatory response, angiogenesis, epithelial–mesenchymal transition and TGFβ signalling in macrophages in the bifurcate portion (Figure 5F). Gene ontology (GO) analysis revealed that in the bifurcate portion, M2‐like macrophages were enriched in immune response activation and lymphocyte proliferation, while M1‐like macrophages showed upregulated lipopolysaccharide response and cytokine production (Figure 5G). Immunofluorescence staining for plasminogen activator, urokinase receptor, urokinase receptor (PLAUR), a receptor involved in plasminogen activation and extracellular matrix degradation, revealed its localisation around macrophages in the bifurcate region (Figure 5H).

**FIGURE 5:**
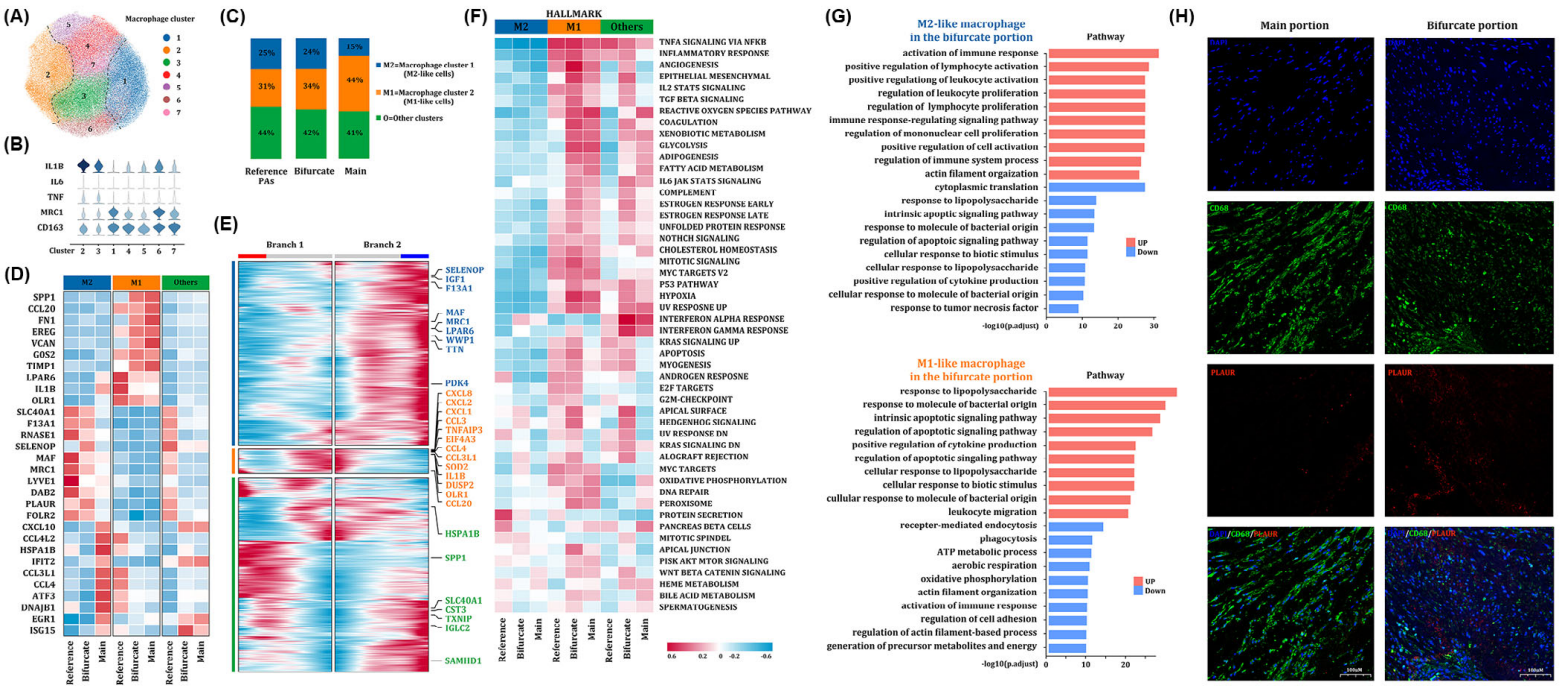
Heterogeneity of macrophage in pulmonary endarterectomised tissues from patients with chronic thromboembolic pulmonary hypertension. (A) Uniform Manifold Approximation and Projection (UMAP) displaying major seven distinct macrophage clusters. (B) Violin plots of depicting top makers for each cluster. (C) Histogram showing the percentage of three kinds of macrophage cell type. (D) Heat map illustrating gene expression levels of the top 10 differentially expressed genes in three macrophage cell type in anatomical regions. (E) Heatmap of the top 15 differentially expressed genes in each branch along the pseudotime trajectory. (F) Heat map enriching the differentially expressed genes in three macrophage cell type in anatomical regions based on the HALLMARK database. (G) Gene ontology (GO) terms (molecular function) analysis of enriched genes in the bifurcate portion compared with the corresponding reference pulmonary arteries (PAs; in the main portion). (H) Immunofluorescence with PLAUR and CD68 in the bifurcate and main portions. PLAUR, plasminogen activator, urokinase receptor.

### 2.5 Smooth muscle cells transition to myofibroblasts in CTEPH

We identified seven distinct SMC clusters (SMCs_1–5), one fibroblast cluster and one myofibroblast cluster (Figure 6A,B). In CTEPH, the proportion of myofibroblasts and fibroblasts was markedly increased, while SMCs were significantly decreased (Figure 6C). DEGs analysis revealed considerable heterogeneity within the SMC clusters, with shared fibroblast markers such as *POSTN*, *FN1*, *COL1A1* and *LUM* (Figure 6D). Pseudotime analysis indicated a trajectory starting at SMCs, progressing through fibroblasts to myofibroblasts (Figure S11). Along this trajectory, a significant transition from SMCs_2 to fibroblasts and myofibroblasts was observed in CTEPH (Figure 6E). The SMCs_2 subcluster exhibited the most significant changes over pseudotime, gaining fibroblast markers (*LUM*, *FN1*, *POSTN*) in CTEPH (Figure 6F). RNA velocity analysis confirmed a differentiation continuum from SMCs_2 to myofibroblasts in CTEPH, which was not observed in reference PAs (Figures 6G and S12). GO analysis of DEGs in SMCs_2 revealed upregulation of extracellular matrix organisation and extracellular structure organisation, while vasculature development, angiogenesis and SMC proliferation were downregulated (Figure 6H). Immunofluorescence staining confirmed the expression of fibroblast markers (LUM and POSTN) in SMCs, with increased expression and co‐localisation observed more prominently in the bifurcate portion compared to the main portion (Figure 6I).

**FIGURE 6:**
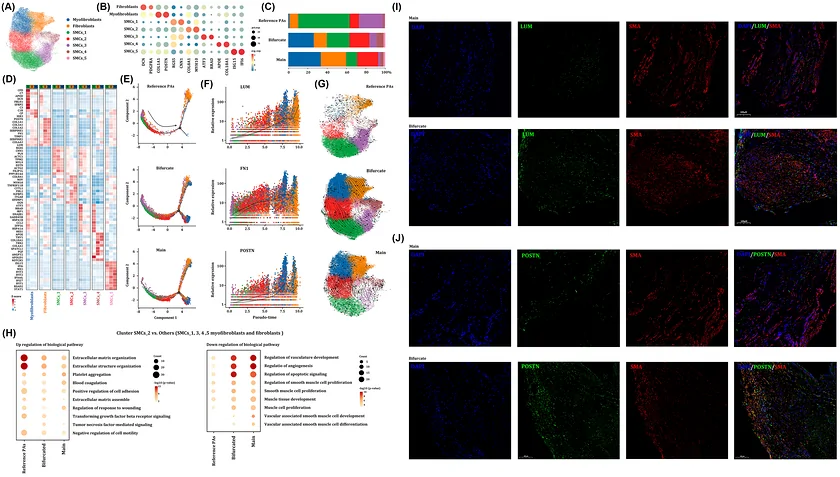
Analysis of smooth muscle cells transition to myofibroblasts in pulmonary endarterectomised tissues from patients with chronic thromboembolic pulmonary hypertension (CTEPH). (A) Uniform Manifold Approximation and Projection (UMAP) displaying major five distinct smooth muscle cells (SMCs) clusters, one fibroblast cluster and one myofibroblast cluster. (B) Top markers for each subcluster. (C) Horizontal slice bar representing the anatomical distribution of each identified cell type. (D) Heat map displaying gene expression levels of the top 10 differentially expressed genes in each cluster. (E) Pseudotime trajectory from the SMCs subcluster to myofibroblasts subcluster in CTEPH tissues. (F) Increased expressions of myofibroblasts markers VIM, FN1 and POSNT in CTEPH along the pseudotime trajectory. (G) UMAP embedding of RNA velocity stream presenting the spectrum of SMCs transition to myofibroblasts. (H) Gene ontology (GO) pathway analysis of differentially expressed genes (DEGs; upregulated and downregulated genes in SMCs_2 subcluster) comparison with these of other clusters (SMCs_1, 3, 4, 5, myofibroblast and fibroblasts) in different anatomical portions. (I) Immunofluorescence with LUM, POSNT and SMA in the bifurcate and main portions. LUM, lumican; POSNT, periostin.

## 3 DISCUSSION

Surgical removal of PA obstructions can effectively cure CTEPH in some patients; however, many are not surgical candidates. This study provides a comprehensive cellular composition of CTEPH and sheds light on gene expression alteration across different anatomical regions. Our findings reveal distinct cellular differences between the main and its bifurcate portions, including SMCs transition to myofibroblasts, and macrophages and T‐cell driving inflammation. These results suggest that removal of proximal occlusion without endarterectomy of all the distal branches would leave significant disease behind for CTEPH.

Previous studies have described the heterogeneous cell populations, such as intimal sarcoma like cells,^16^ active myofibroblast‐like cells^17^ and mesenchymal progenitor cells,^18^ but did not differentiate between the main and subsegmental portions. These studies are based on limited markers staining, which may have missed additional cell populations, abundant macrophage (CD68), neutrophils, T‐lymphocytes and angiostatic factors, contributing to inflammatory accumulation and impaired angiogenesis.^19^, ^20^ However, our study clearly identified specific subsets of CD8 Teff and NK cells contributing to inflammatory response, whereas naïve T cells promoted immune dysregulation in the main portion.

Pathological changes of different anatomical regions are significantly different. The main portions exhibited pronounced muscularised and fibrotic thrombi organisation,^7^, ^8^, ^11^ while distal (lobar and segmental) PA obstructions appear as atheromatous lesions or colander‐like lesions.^3^, ^12^, ^21^ Small muscular PA (100–500 µM in diameter) remodelling are similar to those observed in IPAH, such as intimal thickening, fibrosis, fibromuscular proliferation and plexiform lesions.^2^, ^11^, ^22^ Despite yellow fibrotic clots were not easily visible in bifurcate regions, the altered cellular landscape is similar with those in the main portion. PEA surgery can reliably tackle disease in the main PA and its lobar branches, however, distal or micro‐vessels remodelling remains.^11^, ^23^ Whether PH‐targeted medical therapy play a role after PEA in operable patients need to explore.

In this study, compared to the bifurcate portion, a significant decrease of T and NK cells in the main portion was observed. Specifically, CD8^+^ Teff cluster exhibited the activation of inflammatory response in the bifurcate portion, whereas naïve T cells in the main portion contributed to immune dysregulation. These findings are consistent with previous studies linking T cells to chronic inflammation in thrombotic lesions.^13^, ^14^ However, unlike prior studies that treated PEA material as a single homogeneous entity, our study introduces a novel intra‐individual regional comparison, revealing that distinct anatomical zones harbour opposing T cells functional states, and the spatial heterogeneity previously masked in pooled analysis. The activation of naïve CD8^+^ T cells can lead to differentiation into effector (Teff) populations, if antigen is cleared, form memory T cells capable of rapid recall responses.^24^, ^25^ During chronic conditions, T cells may become exhausted, potentially promoting disease progression.^26^, ^27^ The recruitment of CD8^+^ Teff cells also triggers the expression of chemokines and cytokines, which further attract endogenous lymphocytes.^28^, ^29^ In the bifurcate portion, CD8^+^ Teff cells maintained their role in inflammatory recruitment, while naïve T cells were more prevalent, suggesting their role in initiating inflammation and vascular remodelling, thereby indicating that naïve T cells in the bifurcate region as a therapeutic target.

We identified two distinct macrophage populations: one with high IL1B expression, indicative of an M1‐like phenotype, and another characterised by high MRC1 and CD163, typical of M2‐like macrophages.^30^ Although the M1/M2 classification is an oversimplification, it is widely used to describe macrophage diversity, with M1 being pro‐inflammatory and M2 anti‐inflammatory.^31^, ^32^ Pseudotime analysis revealed an increase in M1‐like macrophages and a steady proportion of M2‐like macrophages. Interestingly, the expression of PLAUR, a key regulator of macrophage adhesion and migration, was significantly reduced in the bifurcate portion, indicating a potential mechanism for macrophage infiltration and vascular wall remodelling.^13^, ^33^, ^34^ This highlights the need to explore macrophage phenotypic diversity in CTEPH pathogenesis using advanced technologies like scRNA‐seq.

SMCs exhibited significant heterogeneity in CTEPH, which was similar with observation in atherosclerosis lesion.^13^, ^35^, ^36^ These cells transition from a contractile to a secretory phenotype, and produce excessive extracellular matrix. In our study, a specific cluster of SMCs gained myofibroblast markers, including VIM, POSTN and FN1, and RNA velocity analysis suggested a phenotypic continuum from SMCs to myofibroblasts. This transition was associated with a loss of contractile features, as revealed by GO analysis. Myofibroblast formation in CTEPH may play a role in the organisation of thrombotic materials.^18^, ^29^, ^37^ In the bifurcate portion, the presence of inflammatory T cells and macrophages promote SMCs' transformation into myofibroblasts, suggesting that targeting SMC phenotypic switching pathways might be a relevant avenue for future CTEPH therapeutic exploration.

### 3.1 Limitations of the study

First, we focused on lesions in the main and segmental/subsegmental PAs, which may not fully reflect the biology of smaller vessels. Second, we separated the main and bifurcate portions according to anatomical regions, however, we were unable to normalise the lesion data by specimen slice number. Due to the limited cohort size, an independent validation cohort is absent, and the associations between regional cellular heterogeneity and preoperative haemodynamics, treatment response or post‐PEA prognosis remain to be elucidated. While scRNA‐seq allowed us to identify specific genes in different cell types, further functional analyses and spatial validation (spatial transcriptomics, multiplex imaging) are needed to clarify in situ organisation and mechanistic role of these cell clusters. Additionally, no interventional animal studies or in vitro functional assays were performed; therefore, the causal roles of identified pathways remain speculative. Finally, the study did not correlate cellular profiles utility is not yet established.

In conclusion, this study characterised the anatomical cellular heterogeneity of CTEPH, utilising scRNA‐seq to explore the heterogeneity of immune and vascular cells in different anatomical regions. Our findings demonstrate that the main and bifurcate portions of CTEPH lesions exhibit distinct cellular compositions, with T and NK cells, macrophages and SMCs playing critical roles in inflammation and vascular remodelling. These results highlight the complexity of CTEPH pathogenesis and suggest that targeting specific cellular pathways, such as the SMC‐to‐myofibroblast transition and T‐cell recruitment, could offer novel therapeutic opportunities.

## 4 METHODS

### 4.1 Specimen collection and clinical characteristics of patients

Ten patients with CTEPH undergoing PEA surgery at Shanghai Pulmonary Hospital were enrolled in an ongoing clinical study approved by the Shanghai Pulmonary Hospital Ethics Committee (Number: K18‐055) and conducted according to the principles of the Declaration of Helsinki. Exclusion criteria included patients younger than 18 years or those unable to provide informed consent. PEA samples were collected, including the main/lobar and its bifurcate portions (Figure 1A). The demographic and clinical characteristics of all patients are summarised in Table S2, with preoperative imaging (CT angiogram of chest and VQ scan) shown in Figure S13. Additionally, six reference distal PAs were obtained via surgical lobectomy for peripheral small pulmonary nodules (not for lung cancer). Written inform consent was obtained from both patients and reference subjects.

### 4.2 Sample collection and preparation

Patients were transitioned to cardiopulmonary bypass and the specimens were extracted during deep hypothermic circulatory arrest. After the fresh and visible thrombus removed, portions of the main/lobar/segmental and its bifurcate were dissected and preserved in tissue solution on ice. The materials removed at the time of surgery are partially shown in Figure S14. Six reference PAs (segmental/subsegmental arteries of 30–10 mm in diameter) were obtained during surgical lobectomy as non‐diseased reference tissues to establish baseline cell‐type annotation and marker expression. All fibrothrombotic materials and reference PAs were isolated for scRNAseq. Only nine samples of main portion were used, because there was one case that, for unknown reasons, failed the quality control test. Canonical markers and corresponding cell types were listed in Table S3.

### 4.3 Tissue single‐cell dissociation

Within 30 min post‐surgery, fresh tissue samples were placed into ice‐cold sCelLive® Tissue Preservation Solution (Singleron). The specimens were rinsed three times with Hanks Balanced Salt Solution (HBSS), followed by mechanical fragmentation and enzymatic digestion using Tissue Dissociation Solution (Singleron) for 15 min at 37°C. Cell homogenate was filtered through a 40‐micron sterile strainer. To eliminate erythrocytes, GEXSCOPE® lysis buffer (RCLB, Singleron) was added at 1:2 (v/v) ratio of cell to buffer and incubated at room temperature for 5–8 min. We centrifuged at 300 × *g* (5 min at 4°C), the resulting pellet was gently resuspended. Cell viability was determined under a microscope via Trypan Blue staining.

### 4.4 Single‐cell RNA sequencing

Single‐cell suspensions at 2 × 10^5^ cells/mL were loaded onto microwell chip via the Singleron Matrix® Single Cell Processing System. Barcoding Beads were subsequently harvested and subjected to reverse transcription to capture mRNA. The amplified cDNA products underwent fragmentation via enzymatic digestion, followed by the ligation of sequencing adapters. Libraries preparation was carried out in accordance with GEXSCOPE® Single Cell RNA Library Kits (Singleron).^38^ Normalisation to 4 nM, the barcode libraries were pooled and paired‐end sequencing (150 bp) on Illumina novaseq 6000 platform.

### 4.5 Primary analysis of raw read data

We utilised the CeleScope v1.9.0 pipeline to transform scRNA‐seq raw reads into gene expression matrices (https://github.com/singleron‐RD/CeleScope). In brief, initial quality filtering was conducted by trimming poly‐A and removing adapter contamination using Cutadapt v1.17. Following the extraction of cell barcode and unique molecular identifiers (UMIs), reads were aligned against GRCh38 (ensembl version 92 annotation) employing STAR v2.6.1a.^39^ Ultimately, UMI and gene counts per individual cell were calculated via the feature Counts v2.0.1 software, producing the downstream expression matrices.^40^

### 4.6 Quality control, dimension‐reduction and clustering

Data processing, including quality filtering, dimensionality reduction and clustering identification, was conducted with Scanpy v1.8.2 within a Python 3.7 environment.^41^ For each sample dataset, a filtering pipeline was applied: low‐quality cells were discarded if they expressed fewer than 200 genes or below top 2% gene expression. Cells in the highest top 2% UMI count were removed if exceeding a 30% mitochondrial gene ratio were eliminated. Additionally, genes expressed in less than five cells were omitted. The resulting count matrix underwent normalisation against total counts per cell and subsequent log‐transformation. Using the ‘seurat’ setting, the 2000 most variable genes were identified, followed by principal component analysis (PCA) on the scaled data, with the top 17 principal components driving clustering and reduction. Ultimately, the Louvain algorithm cells into 20 distinct group, visualised via Uniform Manifold Approximation and Projection (UMAP).

### 4.7 Differentially expressed genes analysis

To identify DEGs, we used the scanpy.tl.rank_genes_groups function based on Wilcoxon rank‐sum test with default parameters. The expression in over 10% of cells within at least one of the compared groups, and an average log2 fold change exceeding  .25 was selected as DEGs. To account for multiple testing, the Benjamini–Hochberg method was applied to compute adjusted *p* value, with the value below  .05 was considered significantly.

### 4.8 Cell‐type annotation

Cluster identities were established by mapping their expression profiles against canonical markers curated within the SynEcoSys® database (Singleron Biotechnology), an annotation reference that aggregates established cell‐type signatures from sources including CellMakerDB, PanglaoDB and recent published studies. A detailed inventory of the specific canonical markers utilised for these assignments is provided in Table S3.

### 4.9 Pathway enrichment analysis

Potential biological functions of identified genes were examined via GO enrichment utilising the ‘clusterProfiler’ R package v 4.0.2.^42^ Pathways were deemed significantly enriched when their adjusted *p* value fell below  .05; the leading terms were then depicted as bar plots. For Gene Set Variation Analysis (GSVA), input data consisted mean gene expression calculated for distinct cell types.^43^ Functional annotation was performed using GO gene sets, including molecular function (MF), biological process (BP) and cellular component (CC) categories were used.

### 4.10 Trajectory analysis

We constructed the differentiation trajectory of cells usingMonocle2.^44^ To order cells along the pseudo‐temporal axis, Highly variable genes (HVGs) were selected. DDRTree was then applied to perform dimension‐reduction and identify variable features. The trajectory was ultimately mapped using plot_cell_trajectory tool.

### 4.11 Cell–cell interaction analysis (CellPhoneDB)

Cell–cell communication was inferred via the CellPhoneDB v2.1^45^ leveraging annotated receptor–ligand interactions across cell subset.^46^ To determine background expectations, cell clusters were shuffled 1000 times to calculate the null distribution of average ligand–receptor expression levels. The expression cutoff for each ligand or receptor was determined from the average log‐expression distribution. Interactions with *p* value <.05 and average log expression >.1 were retained as significant. Visualisation of these significant pairs was achieved using v0.4.14 R package.

### 4.12 Transcription factor regulatory network analysis

To reconstruct regulatory network, data of single‐cell expression and transcription factors sourced from AnimalTFDB were processed through pyscenic v0.11.0 pipeline.^46^ The, GRNBoost2 module was initially employed to infer potential interactions based on the co‐expression patterns between regulators and targets. Subsequently, CisTarget was implemented to filter out indirect targets and enriched binding motifs. Regulon activity within individual cells were then quantified using AUCell. Visualisation of the top RSS‐ranked regulons was displayed through pheatmap in R.

### 4.13 UCell gene set scoring

Per‐cell gene set activity was determined via the UCell v 2.2.0 R package.^47^ This tool calculated a score for each cell based on the Mann–Whitney *U* statistic, relying on the relative ranking of query gene. Because this scoring method based on rank order, it is suitable for large‐scale datasets with multiple biological samples or technical batches.

### 4.14 OMIM search for pulmonary arterial diseases

A comprehensive search of the OMIM database was performed using the terms ‘pulm* or arteri* or thrombi*’, yielding 528 entries. These were manually reviewed to identify conditions affecting the PAs, pulmonary vasculature, pulmonary embolism and thrombosis, excluding syndromes caused by chromosomal disorder and contiguous gene deletion. Entries containing terms such as ‘dysplasia’ of bone and spine, ‘pulmonary valve stenosis’ and ‘congenital defect’, but no terms related to primary pulmonary disorders, were also excluded from further consideration (Table S4). The final list was compared to genes identified in the top 50 DEGs per cluster to generate cluster specific marker expression.

### 4.15 Supervised analyses using GWAS genes

Supervised analyses were conducted using GWAS genes associated with PAH. The PAH/CTEPH‐associated GWAS gene list was compiled from the GWAS Catalogue provided by EMBL‐EBI using the search term ‘PAH/CTEPH’ (https://www.ebi.ac.uk/gwas/). The list was retrieved on 30 November 2022 (Table S5). Genes in the top 50 hits from both our single‐cell differential expression marker list and PAH/CTEPH‐associated GWAS list were selected for hierarchical clustering. This clustering was visually represented as a heatmap to facilitate data interpretation and analysis.^48^ DEGs among cell clusters assessed via Wilcoxon rank‐sum tests, multiple testing correction applied using the Benjamini–Hochberg procedure (adjusted *p* < .05).

### 4.16 Tissue collection and histopathological analysis

Samples were fixed with 4% paraformaldehyde, embedded in paraffin and cut at 4 µM thickness. Standard procedures were followed for haematoxylin and eosin (H&E), Masson's trichrome and Elastin van Gieson (EVG) staining. Micrographs were obtained using a Leica DC 500 camera fitted onto a Leica DMRE microscope.^49^ Immunofluorescence was performed using the following primary antibodies: anti‐CD31(ab305267, Abcam), anti‐alpha smooth muscle actin antibody (αSMA, ab7817, Abcam), anti‐fibronectin (66042‐1‐Ig, Proteintech), anti‐lumican antibody (ab168348, Abcam), anti‐periostin antibody (66491‐1‐Ig, Proteintech) and anti‐PLAUR antibody (10286‐1‐AP, Proteintech). Sections were mounted and counterstained with ProLong™ gold antifade reagent containing 4′6‐diamidino‐2‐phenylindole (DAPI, p36931, Invitrogen). Images were captured with a fluorescence microscope and analysed using CaseViewer 2.4 software (3DHISTECH Ltd.).

### 4.17 Statistical analysis

To assess tissue‐level gene expression differences, the Wilcox.test function of R was utilised to run a one‐sample Wilcoxon signed‐rank test, which determined whether the logFC across all genes deviated significantly from zero.

## AUTHOR CONTRIBUTIONS

Rui Zhang: writing – original draft, resources, visualisation, investigation, methodology, visualisation, conceptualisation, supervision, resources, project administration, funding acquisition. Yunlong Zhang: writing – original draft, resources, visualisation, investigation, methodology, visualisation, conceptualisation, supervision, resources, project administration, funding acquisition. Huiting Li: methodology, investigation, formal analysis, data curation. Jing Zhang: validation, methodology, investigation, formal analysis, data curation. Shou Han: visualisation, methodology, investigation. Xinbo Cheng: visualisation, methodology, investigation. Qinhua Zhao: resources, conceptualisation. Jing He: resources, conceptualisation. Cijun Luo: resources, conceptualisation. Hongling Qiu: resources, conceptualisation. Jianfeng Zhang: resources, conceptualisation. Sugang Gong: writing – review and editing, conceptualisation, supervision, resources, project administration. Lan Wang: writing – review and editing, conceptualisation, supervision, resources, project administration, funding acquisition.

## CONFLICT OF INTEREST STATEMENT

The authors declare no conflicts of interest.

## ETHICS STATEMENT

All samples were collected in an ongoing clinical study approved by the Shanghai Pulmonary Hospital Ethics Committee (Number: K18‐055) and conducted according to the principles of the Declaration of Helsinki.

## Novelty and Relevance

This study reveals the comprehensive cellular composition and gene expression landscape of different anatomical regions in chronic thromboembolic pulmonary hypertension (CTEPH) tissues at the single‐cell level.
The regions with colander‐like lesions changes had more obviously T cell and macrophages driving immune‐inflammatory response and smooth muscle cells (SMCs) differentiation.

Relatively little attention has been given to the difference between the main portion and the bifurcate portion in pulmonary endarterectomy (PEA) tissues. In this study, we used single‐cell RNA sequencing technology to investigate cellular types and cell fate across the different anatomical regions contributing to the development of CTEPH.

## Clinical/Pathophysiological Implications?

PEA surgery can reliably tackle disease in the main pulmonary arteries and their lobar branches, however, the remodelling of distal or micro‐vessels still remained. Our findings suggest removal of proximal occlusion without endarterectomy of all the distal branches would leave significant disease behind for CTEPH.

## Supporting information



Supporting Information File 1: ctm270864‐sup‐0001‐SupMat.docx

## Data Availability

The datasets generated during the current study are available in the Genome Sequence Archive (Genomics, Proteomics & Bioinformatics 2025) in National Genomics Data Center (Nucleic Acids Res 2026), China National Center for Bioinformation/Beijing Institute of Genomics, Chinese Academy of Sciences (GSA: HRA017942) that are publicly accessible at https://ngdc.cncb.ac.cn/gsa‐human/submit/hra/subHRA028247.
