# Supplementary material for: A single‐cell census of pulmonary endarterectomies tissue across different anatomical regions in chronic thromboembolic pulmonary hypertension
Source: Clin Transl Med. 2026 Oct 11;16(10):e70864. doi: 10.1002/ctm2.70864 (PMC13655372; doi:10.1002/ctm2.70864)
Supplement: Supplementary file 1 [file CTM2-16-e70864-s001.docx]

**Supplementary tables and figures**

**Supplemental Tables**

**Supplemental Table 1 Top 20 marker genes in each macrophages type.**

| **Names** | **Scores** | **Log fold changes** | **P_vals** | **P vals_adj** | **pct_nz_group** | **pct_nz_reference** | **mean_group** | **mean_reference** | **Cluster** |
| --- | --- | --- | --- | --- | --- | --- | --- | --- | --- |
| IGLC2 | 50.26526 | 2.550804 | 0 | 0 | 0.312668 | 0.078049 | 0.77146 | 0.140089 | M1 |
| IGKC | 38.93946 | 2.361616 | 0 | 0 | 0.411712 | 0.216024 | 1.105946 | 0.384697 | M1 |
| IGHG3 | 32.0429 | 1.921335 | 2.76E-225 | 3.17E-223 | 0.2562 | 0.106624 | 0.548963 | 0.17894 | M1 |
| SLC40A1 | 57.34781 | 1.910539 | 0 | 0 | 0.699432 | 0.416953 | 1.884065 | 0.785936 | M1 |
| IGHG1 | 34.01506 | 1.530688 | 1.33E-253 | 1.81E-251 | 0.22184 | 0.076434 | 0.471854 | 0.124175 | M1 |
| F13A1 | 49.73497 | 1.499039 | 0 | 0 | 0.754108 | 0.569063 | 2.056939 | 1.127887 | M1 |
| IGHG4 | 20.77572 | 1.4151 | 7.18E-96 | 3.77E-94 | 0.27308 | 0.169578 | 0.582469 | 0.298213 | M1 |
| PDK4 | 33.46293 | 1.262589 | 1.67E-245 | 2.12E-243 | 0.461906 | 0.28958 | 1.128405 | 0.53903 | M1 |
| MRC1 | 50.67357 | 1.238232 | 0 | 0 | 0.791754 | 0.628534 | 2.14379 | 1.288028 | M1 |
| SELENOP | 32.91648 | 1.178284 | 1.28E-237 | 1.57E-235 | 0.788019 | 0.705826 | 2.492959 | 1.772509 | M1 |
| TXNIP | 55.09565 | 1.133362 | 0 | 0 | 0.879594 | 0.733794 | 2.462991 | 1.58164 | M1 |
| IGHA1 | 23.10338 | 1.126122 | 4.28E-118 | 2.64E-116 | 0.183597 | 0.086935 | 0.359533 | 0.1348 | M1 |
| CPVL | 44.48672 | 1.122212 | 0 | 0 | 0.634001 | 0.41251 | 1.399232 | 0.715979 | M1 |
| MAF | 45.9081 | 1.114862 | 0 | 0 | 0.728712 | 0.563813 | 1.729391 | 1.016099 | M1 |
| SAMHD1 | 46.08672 | 1.021268 | 0 | 0 | 0.711085 | 0.51454 | 1.549703 | 0.878916 | M1 |
| WWP1 | 33.4329 | 1.003331 | 4.56E-245 | 5.74E-243 | 0.36331 | 0.192902 | 0.701923 | 0.286363 | M1 |
| LPAR6 | 44.59467 | 0.993197 | 0 | 0 | 0.751419 | 0.593901 | 1.728196 | 1.063602 | M1 |
| NRP1 | 38.70492 | 0.987026 | 0 | 0 | 0.640424 | 0.482785 | 1.38789 | 0.812529 | M1 |
| IGLC3 | 19.95091 | 0.982408 | 1.47E-88 | 7.24E-87 | 0.107111 | 0.042609 | 0.213627 | 0.064192 | M1 |
| TTN | 24.11542 | 0.980415 | 1.72E-128 | 1.14E-126 | 0.164924 | 0.070275 | 0.352691 | 0.113448 | M1 |
| CCL20 | 47.7224 | 2.231646 | 0 | 0 | 0.378551 | 0.121734 | 0.96345 | 0.229313 | M2 |
| IL1B | 78.9836 | 2.212759 | 0 | 0 | 0.935619 | 0.519097 | 3.637662 | 1.460791 | M2 |
| G0S2 | 61.37337 | 2.126978 | 0 | 0 | 0.569507 | 0.192624 | 1.547615 | 0.412516 | M2 |
| ZNF331 | 72.78656 | 2.018935 | 0 | 0 | 0.71407 | 0.25933 | 1.739981 | 0.477015 | M2 |
| OLR1 | 80.41888 | 1.968832 | 0 | 0 | 0.812574 | 0.302827 | 1.899709 | 0.544006 | M2 |
| PLAUR | 80.75337 | 1.918238 | 0 | 0 | 0.917297 | 0.498078 | 2.471673 | 0.965821 | M2 |
| C15orf48 | 75.93063 | 1.912631 | 0 | 0 | 0.695243 | 0.20975 | 1.525527 | 0.371114 | M2 |
| BCL2A1 | 80.85551 | 1.849166 | 0 | 0 | 0.901664 | 0.423199 | 2.360398 | 0.844714 | M2 |
| NR4A3 | 81.55056 | 1.765374 | 0 | 0 | 0.815935 | 0.267504 | 1.913137 | 0.531373 | M2 |
| MIR155HG | 66.24121 | 1.563888 | 0 | 0 | 0.57455 | 0.164356 | 1.120533 | 0.275387 | M2 |
| GPR183 | 61.55201 | 1.510532 | 0 | 0 | 0.884014 | 0.568579 | 2.452133 | 1.239864 | M2 |
| INSIG1 | 54.83118 | 1.393853 | 0 | 0 | 0.634056 | 0.277964 | 1.331568 | 0.495862 | M2 |
| NFKB1 | 66.5074 | 1.387554 | 0 | 0 | 0.601446 | 0.179001 | 1.08697 | 0.285813 | M2 |
| VEGFA | 63.8768 | 1.376316 | 0 | 0 | 0.637922 | 0.217486 | 1.234443 | 0.37645 | M2 |
| CCRL2 | 63.80273 | 1.338001 | 0 | 0 | 0.542276 | 0.15516 | 0.946952 | 0.234049 | M2 |
| PHLDA1 | 59.50293 | 1.331798 | 0 | 0 | 0.642125 | 0.24775 | 1.289931 | 0.441978 | M2 |
| MT2A | 37.65185 | 1.309927 | 3.0516299808738e-310 | 5.54E-308 | 0.732896 | 0.504695 | 1.988556 | 1.152039 | M2 |
| PDE4B | 65.37525 | 1.301769 | 0 | 0 | 0.790721 | 0.359656 | 1.617922 | 0.631142 | M2 |
| CCR7 | 38.05387 | 1.277652 | 0 | 0 | 0.135821 | 0.019705 | 0.301157 | 0.03269 | M2 |
| TWISTNB | 46.26046 | 1.256905 | 0 | 0 | 0.449151 | 0.169805 | 0.875704 | 0.29468 | M2 |
| JUN | 69.11948 | 1.174324 | 0 | 0 | 0.952738 | 0.763505 | 2.960614 | 1.89332 | Others |
| CCL8 | 28.23629 | 1.070204 | 2.10E-175 | 1.17E-172 | 0.422325 | 0.269952 | 1.102095 | 0.621882 | Others |
| HSPA1B | 59.22384 | 0.999635 | 0 | 0 | 0.911754 | 0.653801 | 2.719017 | 1.686641 | Others |
| EGR1 | 47.99152 | 0.994156 | 0 | 0 | 0.779133 | 0.538322 | 1.92781 | 1.162493 | Others |
| DNAJB1 | 57.71645 | 0.97769 | 0 | 0 | 0.927484 | 0.703393 | 3.232026 | 2.116828 | Others |
| FOS | 64.75912 | 0.93221 | 0 | 0 | 0.987517 | 0.941944 | 3.918142 | 3.053543 | Others |
| EGR2 | 32.26848 | 0.896647 | 1.94E-228 | 1.52E-225 | 0.370662 | 0.202404 | 0.716665 | 0.341401 | Others |
| HSP90AA1 | 52.5427 | 0.849522 | 0 | 0 | 0.984126 | 0.930871 | 3.896163 | 3.129248 | Others |
| CXCL1 | 27.32317 | 0.8362 | 2.25E-164 | 1.20E-161 | 0.442745 | 0.291545 | 1.160969 | 0.696593 | Others |
| IER5 | 38.15342 | 0.830626 | 0 | 0 | 0.585035 | 0.363047 | 1.144249 | 0.647429 | Others |
| IER2 | 38.89129 | 0.82005 | 0 | 0 | 0.674724 | 0.469667 | 1.39657 | 0.863992 | Others |
| CXCL10 | 7.28992 | 0.785614 | 3.10E-13 | 1.06E-11 | 0.151021 | 0.121411 | 0.3421 | 0.251778 | Others |
| CCL2 | 30.54405 | 0.78242 | 6.78E-205 | 4.81E-202 | 0.726099 | 0.580558 | 2.149217 | 1.541645 | Others |
| DUSP1 | 50.55672 | 0.779732 | 0 | 0 | 0.946244 | 0.795935 | 2.866993 | 2.084627 | Others |
| GADD45B | 49.33746 | 0.777525 | 0 | 0 | 0.912115 | 0.740252 | 2.545959 | 1.816132 | Others |
| HSPB1 | 35.94844 | 0.763293 | 5.35E-283 | 5.17E-280 | 0.822137 | 0.682908 | 2.182576 | 1.603229 | Others |
| HSPA1A | 51.52153 | 0.747834 | 0 | 0 | 0.959665 | 0.808432 | 3.614265 | 2.649853 | Others |
| HSPA6 | 20.62814 | 0.714227 | 1.53E-94 | 5.02E-92 | 0.3887 | 0.278731 | 0.85282 | 0.555097 | Others |
| SGK1 | 43.42045 | 0.714005 | 0 | 0 | 0.909517 | 0.75615 | 2.574233 | 1.90314 | Others |
| ATF3 | 38.84278 | 0.681499 | 0 | 0 | 0.778123 | 0.531915 | 1.987718 | 1.311878 | Others |

| **Supplemental Table 2 Clinical Characteristics of the three subjects in the study** | | | | | | | | | | | |
| --- | --- | --- | --- | --- | --- | --- | --- | --- | --- | --- | --- |
| **Variables** | **Patient**  **1** | **Patient**  **2** | **Patient**  **3** | **Patient**  **4** | **Patient**  **5** | **Patient**  **6** | **Patient**  **7** | **Patient**  **8** | **Patient**  **9** | **Patient**  **10** | **Patient**  **11** |
| Age, years | 65 | 62 | 51 | 73 | 54 | 55 | 52 | 60 | 65 | 65 | 70 |
| Gender | Female | Female | Male | Female | Male | Male | Male | Female | Female | Female | Female |
| Weight, kg | 45 | 52 | 75 | 72 | 68 | 70 | 72 | 50 | 55 | 50 | 47 |
| WHO FC | III | III | III | III | III | III | III | III | III | III | III |
| Time from first thromboembolic event to diagnosis, years | 17 | 5 | 4 | 2 | 4 | 5 | 5 | 3 | 4 | 6 | 12 |
| NT-proBNP, pg/ml | 1878 | 2545 | 1278 | 1863 | 2855 | 1225 | 2198 | 1987 | 1824 | 2005 | 2430 |
| D-Dimer, ng/mL | 308 | 668 | 734 | 338 | 276 | 313 | 603 | 352 | 289 | 277 | 660 |
| **Right heart catheterization** | | | | | | | | | | | |
| RAP, mmHg | 2 | 2 | 5 | 3 | 5 | 5 | 4 | 3 | 2 | 3 | 2 |
| mPAP, mmHg | 59 | 64 | 46 | 39 | 48 | 45 | 50 | 56 | 55 | 55 | 60 |
| PAWP, mmHg | 7 | 5 | 2 | 5 | 4 | 3 | 3 | 4 | 4 | 4 | 5 |
| CO, L/min | 3.3 | 3.2 | 3.2 | 3.7 | 2.9 | 3.2 | 3.4 | 3.3 | 3.3 | 3.2 | 3.0 |
| CI, L/min/m^2^ | 2.4 | 2.1 | 1.6 | 2.0 | 2.0 | 2.3 | 2.4 | 2.1 | 2.2 | 2.3 | 2.1 |
| PVR, Wood units | 13.8 | 18.27 | 13.8 | 11.5 | 12.7 | 11.52 | 13.87 | 12.24 | 13.6 | 13.8 | 14.34 |
| **Scintigraphy and CT angiography, % abnormal** | 100% | 100% | 100% | 100% | 100% | 100% | 100% | 100% | 100% | 100% | 100% |
| **History of infected lines** | No | No | No | No | No | No | No | No | No | No | No |
| **History of splenectomy** | No | No | No | No | No | No | No | No | No | No | No |
| **Inflammatory bowel disease** | No | No | No | No | No | No | No | No | No | No | No |
| **Active cancer/history of cancer** | No | No | No | No | No | No | No | No | No | No | No |
| **Thyroid disease or hormone replacement therapy** | No | No | No | No | No | No | No | No | No | No | No |
| **History of acute venous thromboembolish** | No | Yes | No | No | No | No | No | Yes | Yes | No | No |
| **Anticoagulation** | Warfarin | Rivaroxaban | Rivaroxaban | No oral anticoagulants | Warfarin | Warfarin | Warfarin | Rivaroxaban | Rivaroxaban | Rivaroxaban | Warfarin |
|  |  |  |  |  |  |  |  |  |  |  |  |
| **Medical therapy, %** | | | | | | | | | | | |
| PDE5i | 50% |  |  |  |  |  |  |  |  | 50% | 50% |
| ERA | 50% |  | 50% |  |  |  |  | 50% |  | 50% | 50% |
| sGC |  | 100% | 50% | 100% | 100% | 100% | 100% | 50% | 100% |  |  |

Abbreviations: CI: cardiac index; CO: Cardiac output; ERA: Endothelin receptor antagonist; mPAP: mean pulmonary arterial pressure; NT-proBNP: N-terminal fragmental of pro-brain natriuretic peptide; PAWP: pulmonary artery wedge pressure; PVR: pulmonary vascular resistance; RAP: right atrial pressure; WHO FC: World Health Organization functional class;

**Supplemental Table 3** **Cluster and its corresponding main cell type**

| **Cell type** | **Abbreviation** | **Markers** | **Remarks** |
| --- | --- | --- | --- |
| Endothelial cells | ECs | CDH5, PECAM1, VWF, CLDN5 |  |
| Smooth muscle cells | SMCs | ACTA2, TAGLN, MYL9, MYH11 |  |
| Fibroblasts | Fibroblasts | DCN, LUM, COL1A2 |  |
| T cells and NK cells | T and NK | CD2, CD3D, TRAC, TRBC2 |  |
| Mononuclear phagocytes | MPs | LYZ, CD14, FCN1, MRC1, C1QC, CD1C, XCR1 | including a small number of Neutrophils |
| Mast cells | Mast Cells | TPSAB1, TPSB2, CPA3 |  |
| Plasma cells | Plasma Cells | CD79A, CD79B, JCHAIN, MZB1 | including a small number of B cells |
| Proliferating cells | Proliferating Cells | TOP2A, MKI67 | including a small number of T cells, NK and MPs |
| Natural killer cells | NK | KLRC1, KLRD1, XCL1, NKG7, CD3D- |  |
| CD8+ effector T cells | CD8Teff | CD8A, GZMK, GZMH, NKG7 |  |
| Naive T cells | Naïve T | CCR7, SELL, IL7R, LEF1 | including a small number of T helper cells |
| Proliferating T cells | Proliferating T | TOP2A, MKI67, CD3D |  |
| Monocytes | Monocytes | CD14, VCAN, FCN1, FCGR3A |  |
| Macrophages | Macrophages | MRC1, CD163, C1QC |  |
| Conventional type 2 dendritic cells | cDC2 | CD1C, CD1E, FCER1A |  |
| Conventional type 1 dendritic cells | cDC1 | XCR1, CLEC9A, IDO1 |  |
| Neutrophils | Neutrophils | CSF3R, CXCR2, FCGR3B |  |

**Supplemental Table 4 Clinical Synopsis Search - 'pulm* or arteri* or thrombi* (Search in: Entries with: Molecular basis)'**

| Downloaded: Nov 28, 2022 | | | | | | | |
| --- | --- | --- | --- | --- | --- | --- | --- |
| Copyright (c) 1966-2022 Johns Hopkins University OMIM, data are provided for research purposes only. | | | | | | | |
| **MIM**  **Number** | **Number** | **Title** | **Location** | **Inheritance** | **Gene** | **Phenotype**  **mapping key** | **OMIM**  **number** |
| #147891 | 1 | ISCHIOCOXOPODOPATELLAR SYNDROME WITH OR WITHOUT PULMONARY ARTERIAL HYPERTENSION; ICPPS | 17q23.2 | AD | TBX4 | 3 | 601719 |
| #614224 | 2 | RETINAL ARTERIAL MACROANEURYSM WITH SUPRAVALVULAR PULMONIC STENOSIS; RAMSVPS | 4q12 | AR | IGFBP7 | 3 | 602867 |
| #188050 | 3 | THROMBOPHILIA DUE TO THROMBIN DEFECT; THPH1 | 1p36.22/6p25.1/  10q25.3/11p11.2 | AD | MTHFR/  F13A1/  HABP2/F2 | 3 | 607093/134570/  603924/176930 |
| #208050 | 4 | ARTERIAL TORTUOSITY SYNDROME; ATORS | 20q13.12 | AR | SLC2A10 | 3 | 606145 |
| #178600 | 5 | PULMONARY HYPERTENSION, PRIMARY, 1; PPH1 | 2q33.1-q33.2 | AD | BMPR2 | 3 | 600799 |
| #234810 | 6 | PULMONARY VENOOCCLUSIVE DISEASE 2, AUTOSOMAL RECESSIVE; PVOD2 | 15q15.1 | AR | EIF2AK4 | 3 | 609280 |
| #265380 | 7 | ALVEOLAR CAPILLARY DYSPLASIA WITH MISALIGNMENT OF PULMONARY VEINS; ACDMPV | 16q24.1 | AD | FOXF1 | 3 | 601089 |
| #265450 | 8 | PULMONARY VENOOCCLUSIVE DISEASE 1, AUTOSOMAL DOMINANT; PVOD1 | 2q33.1-q33.2 | AD | BMPR2 | 3 | 600799 |
| #615344 | 9 | PULMONARY HYPERTENSION, PRIMARY, 4; PPH4 | 2q23.3 | AD | KCNK3 | 3 | 603220 |
| #208000 | 10 | ARTERIAL CALCIFICATION, GENERALIZED, OF INFANCY, 1; GACI1 | 6q23.2 | AR | ENPP1 | 3 | 173335 |
| #108010 | 11 | ARTERIOVENOUS MALFORMATIONS OF THE BRAIN | 7q15.3/12p12.1 | Smu | IL6/KRAS | 3 | 147620/190070 |
| #125310 | 12 | CEREBRAL ARTERIOPATHY, AUTOSOMAL DOMINANT, WITH SUBCORTICAL INFARCTS AND LEUKOENCEPHALOPATHY, TYPE 1; CADASIL1 | 19p13.12 | AD | NOTCH3 | 3 | 600276 |
| #600142 | 13 | CEREBRAL ARTERIOPATHY, AUTOSOMAL RECESSIVE, WITH SUBCORTICAL INFARCTS AND LEUKOENCEPHALOPATHY; CARASIL | 10q26.13 | AD | HTRA1 | 3 | 602194 |
| #601360 | 14 | AMELIA, POSTERIOR, WITH PELVIC AND PULMONARY HYPOPLASIA SYNDROME; PAPPAS | 17q23.2 | AD | TBX4 | 3 | 601719 |
| #265100 | 15 | PULMONARY ALVEOLAR MICROLITHIASIS; PULAM | 4p15.2 | AR | SLC234A2 | 3 | 604217 |
| #265120 | 16 | SURFACTANT METABOLISM DYSFUNCTION, PULMONARY, 1; SMDP1 | 2q11.2 | AR | SFTPB | 3 | 178640 |
| #265400 | 17 | PULMONARY HYPERTENSION, PRIMARY, 5; PPH5 | 3q29 | AR | ATP13A3 | 3 | 610232 |
| #618042 | 18 | IMMUNODEFICIENCY 100 WITH PULMONARY ALVEOLAR PROTEINOSIS AND HYPOGAMMAGLOBULINEMIA; IMD100 | 12q24.12 | AD | OAS1 | 3 | 164350 |
| #618196 | 19 | CAPILLARY MALFORMATION-ARTERIOVENOUS MALFORMATION 2; CMAVM2 | 7q22.1 | AD | EPHB4 | 3 | 600011 |
| #618674 | 20 | PULMONARY FIBROSIS AND/OR BONE MARROW FAILURE, TELOMERE-RELATED, 5; PFBMFT5 | 12q24.31 | AD | ZCCHC8 | 3 | 616381 |
| #615342 | 21 | PULMONARY HYPERTENSION, PRIMARY, 2; PPH2 | 13q13.3 | AD | MADH9 | 3 | 603295 |
| #615343 | 22 | PULMONARY HYPERTENSION, PRIMARY, 3; PPH3 | 7q31.2 | AD | CAV1 | 3 | 601047 |
| #615704 | 23 | POIKILODERMA, HEREDITARY FIBROSING, WITH TENDON CONTRACTURES, MYOPATHY, AND PULMONARY FIBROSIS; POIKTMP | 11q12.1 | AD | FAM111B | 3 | 615584 |
| #616371 | 24 | PULMONARY FIBROSIS AND/OR BONE MARROW FAILURE, TELOMERE-RELATED, 4; PFBMFT4 | 16p13.12 | AD | PARN | 3 | 604212 |
| #616373 | 25 | PULMONARY FIBROSIS AND/OR BONE MARROW FAILURE, TELOMERE-RELATED, 3; PFBMFT3 | 20q13.33 | AD | RTEL1 | 3 | 608833 |
| #616779 | 26 | CEREBRAL ARTERIOPATHY, AUTOSOMAL DOMINANT, WITH SUBCORTICAL INFARCTS AND LEUKOENCEPHALOPATHY, TYPE 2; CADASIL2 | 10q26.13 | AD | HTRA1 | 3 | 602194 |
| #617035 | 27 | PATENT DUCTUS ARTERIOSUS 2; PDA2 | 6p12.3 | AD | TFAP2B | 3 | 601601 |
| #617039 | 28 | PATENT DUCTUS ARTERIOSUS 3; PDA3 | 5q23.2 | AD | PRDM6 | 3 | 616982 |
| #613845 | 29 | HYPERURICEMIA, PULMONARY HYPERTENSION, RENAL FAILURE, AND ALKALOSIS SYNDROME; HUPRAS | 19q13.2 | AD | SARS2 | 3 | 612804 |
| #614370 | 30 | SURFACTANT METABOLISM DYSFUNCTION, PULMONARY, 5; SMDP5 | 22q12.3 | AR | CSF2RB | 3 | 138981 |
| #614473 | 31 | ARTERIAL CALCIFICATION, GENERALIZED, OF INFANCY, 2; GACI2 | 16p13.11 | AR | ABCC6 | 3 | 603234 |
| #614742 | 32 | PULMONARY FIBROSIS AND/OR BONE MARROW FAILURE, TELOMERE-RELATED, 1; PFBMFT1 | 5p15.33 | AD | TERT | 3 | 187270 |
| #614743 | 33 | PULMONARY FIBROSIS AND/OR BONE MARROW FAILURE, TELOMERE-RELATED, 2; PFBMFT2 | 3q26.2 | AD | TERC | 3 | 602322 |
| #610913 | 34 | SURFACTANT METABOLISM DYSFUNCTION, PULMONARY, 2; SMDP2 | 8p21.3 | AD | SFTPC | 3 | 178620 |
| #610921 | 35 | SURFACTANT METABOLISM DYSFUNCTION, PULMONARY, 3; SMDP3 | 16q13.3 | AR | ABCA3 | 3 | 601615 |
| #610978 | 36 | CHOREOATHETOSIS AND CONGENITAL HYPOTHYROIDISM WITH OR WITHOUT PULMONARY DYSFUNCTION; CAHTP | 14q13.3 | AD | NKX2-1 | 3 | 600635 |
| #612394 | 37 | BONE FRAGILITY WITH CONTRACTURES, ARTERIAL RUPTURE, AND DEAFNESS; BCARD | 7q22.1 | AD | PLOD3 | 3 | 603066 |
| #608354 | 38 | CAPILLARY MALFORMATION-ARTERIOVENOUS MALFORMATION 1; CMAVM1 | 5q14.3 | AD | RASA1 | 3 | 139150 |
| #619767 | 39 | PULMONARY FIBROSIS AND/OR BONE MARROW FAILURE, TELOMERE-RELATED, 6; PFBMFT6 | 17p13.3 | AD | RPA1 | 3 | 179835 |
| #179613 | 40 | RECOMBINANT CHROMOSOME 8 SYNDROME | 8q22.1 |  |  |  |  |
| #187300 | 41 | TELANGIECTASIA, HEREDITARY HEMORRHAGIC, TYPE 1; HHT1 | 9q34.11 | AD | ENG | 3 | 131195 |
| #201000 | 42 | CARPENTER SYNDROME 1; CRPT1 | 6p12.1-p11.2 | AR | RAB23 | 3 | 606144 |
| #214100 | 43 | PEROXISOME BIOGENESIS DISORDER 1A (ZELLWEGER); PBD1A | 7q21.2 | AR | PEX1 | 3 | 602136 |
| #214800 | 44 | CHARGE SYNDROME | 8q12.2 | AD | CHD7 | 3 | 608892 |
| #300166 | 45 | MICROPHTHALMIA, SYNDROMIC 2; MCOPS2 | Xp11.4 | XLD | BCOR | 3 | 300485 |
| #301054 | 46 | VEXAS SYNDROME; VEXAS | Xp11.3 | XLD | UBA1 | 3 | 314370 |
| #301068 | 47 | HARDIKAR SYNDROME; HDKR | Xq13.1 | XLD | MED12 | 3 | 300188 |
| #306955 | 48 | HETEROTAXY, VISCERAL, 1, X-LINKED; HTX1 | Xq26.3 | XLR | ZIC3 | 3 | 300265 |
| #163950 | 49 | NOONAN SYNDROME 1; NS1 | 12q24.13 | AD | PTPN11 | 3 | 176876 |
| #175050 | 50 | JUVENILE POLYPOSIS/HEREDITARY HEMORRHAGIC TELANGIECTASIA SYNDROME; JPHT | 18q21.2 | AD | SMAD4 | 3 | 600933 |

We applied the search criteria ‘pulm* or arteri* or thrombi*’ and retrieved 528 entries. Here, we showed only 50 terms.

**Supplemental Table 5 Efotraits_EFO_0001361-associations-GWAS**

| **Variant and risk allele** | **p-vale** | **RAF** | **OR** | **CI** | **Mapped gene** | **Reported trait** | **Trait(s)** | **Study accession** | **Location** |
| --- | --- | --- | --- | --- | --- | --- | --- | --- | --- |
| rs71427857-<b>A</b> | 8Χ10-9 | 0.042 | 5.759 | '- | DNAJC10 | Pulmonary arterial hypertension | pulmonary arterial hypertension | GCST004947 | 2:182633113 |
| rs2217560-<b>G</b> | 7Χ10-10 | 0.069 | 1.97 | [1.59-2.45] | CBLN2 | Pulmonary arterial hypertension (without BMPR2 mutations) | pulmonary arterial hypertension | GCST001908 | 18:72483704 |
| rs2856830-<b>C</b> | 8Χ10-20 | 0.12 | 1.56 | [1.42-1.71] | HLA-DPA1 | Pulmonary arterial hypertension | pulmonary arterial hypertension | GCST007228 | 6:33073957 |
| rs13266183-<b>C</b> | 2Χ10-12 | 0.73 | 1.36 | [1.25-1.48] | RNU105C, RN7SL250P | Pulmonary arterial hypertension | pulmonary arterial hypertension | GCST007228 | 8:54355052 |
| rs10103692-<b>G</b> | 5Χ10-15 | 0.9 | 1.8 | [1.55-2.08] | RN7SL250P, RNU105C | Pulmonary arterial hypertension | pulmonary arterial hypertension | GCST007228 | 8:54345567 |

**Supplemental Figure 1** UMAP analysis of all cells with color indicated unbiased classification via graph-based clustering. Cells with similar transcriptional profiles were clustered into 25 different groups, as indicated by the different color. Each dot represents an individual cell.

**Supplemental Figure 2** Heat map represents scaled expression of discriminative gene sets for each cell type. Cell type is summary in columns and genes are shown in rows ranked by adjusted p value< 0.05.

**Supplemental Figure 3** Heat map depicting the number of all possible interactions between the clusters analyzed in different anatomical regions.

**Supplemental Figure 4** Fluorescence staining of pulmonary endarterectomized tissues in the main portion, bifurcate portion and control pulmonary artery. DAPI (nuclei, blue), SMA (green) and CD31 (red) were co-stained. DAPI: 4',6-Diamidino-2-phenylindole, dihydrochloride; SMA: α-smooth muscle actin;

**Supplemental Figure 5** Top 10 marker genes expression in the immune regions.

**Supplemental Figure 6** The differentially expressed genes in the immune regions.

**Supplemental Figure 7** Pseudotime analysis in the immune regions. (**A**) Ordering of cells from different cell types along the pseudotime trajectory. (**B**) Pseudotime value from different cell types along the pseudotime trajectory. (**C**) Pseudotime-ordered analysis of immune cells from patients and healthy control samples. (**D**) The distribution of each cell type across the different branches.

**Supplemental Figure 8** Identified marker genes expression in the T and NK cells.

**Supplemental Figure 9** Top 10 marker genes expression in the T and NK cells.

**Supplemental Figure 10** Circo plots depicting the ligand-receptor interactions among different cell types. (**A**) chemokine type. (**B**) checkpoint type. (**C**) other types.

**Supplemental Figure 11** Pseudotime analysis in the smooth muscle cells and fibroblasts. (**A**) Ordering of cells from different cell types along the pseudotime trajectory. (**B**) Pseudotime value from different cell types along the pseudotime trajectory. (**C**) Pseudotime-ordered analysis from patients and healthy control samples. (**D**) The distribution of each cell type across the different branches.

**Supplemental Figure 12** UMAP embedding of RNA velocity stream presenting the spectrum of SMCs transition to myofibroblasts in CTEPH.

**Supplemental Figure 13** Chest computed tomography angiography (CTA) and ventilation-perfusion (VQ) scan from three patients in this study.

**Supplemental Figure 2** Pictures of fibrothrombotic material removed at the time of pulmonary endarterectomy. According to different anatomical regions, the main and their bifurcate portion were divided. White arrows represent the main portion, and yellow arrows represent their bifurcate portion.
